# Quantification of assembly forces during creation of head-neck taper junction considering soft tissue bearing: a biomechanical study

**DOI:** 10.1186/s42836-021-00075-7

**Published:** 2021-05-01

**Authors:** Toni Wendler, Torsten Prietzel, Robert Möbius, Jean-Pierre Fischer, Andreas Roth, Dirk Zajonz

**Affiliations:** 1 ZESBO - Centre for Research on Musculoskeletal Systems, Leipzig University, Semmelweisstraße 14, 04103 Leipzig, Germany; 2 Institute of Anatomy, Leipzig University, Leipzig, Germany; 3 Department of Orthopaedics, Trauma and Reconstructive Surgery, Zeisigwaldkliniken Bethanien, Chemnitz, Germany; 4 Department of Neurosurgery, Leipzig University, Leipzig, Germany; 5 Department of Orthopaedic, Trauma and Plastic Surgery, Leipzig University, Leipzig, Germany

**Keywords:** Assembly force, Head‐neck taper junction, Soft tissue, Total hip arthroplasty, Biomechanical study

## Abstract

**Background:**

All current total hip arthroplasty (THA) systems are modular in design. Only during the operation femoral head and stem get connected by a Morse taper junction. The junction is realized by hammer blows from the surgeon. Decisive for the junction strength is the maximum force acting once in the direction of the neck axis, which is mainly influenced by the applied impulse and surrounding soft tissues. This leads to large differences in assembly forces between the surgeries. This study aimed to quantify the assembly forces of different surgeons under influence of surrounding soft tissue.

**Methods:**

First, a measuring system, consisting of a prosthesis and a hammer, was developed. Both components are equipped with a piezoelectric force sensor. Initially, *in situ* experiments on human cadavers were carried out using this system in order to determine the actual assembly forces and to characterize the influence of human soft tissues. Afterwards, an *in vitro* model in the form of an artificial femur (Sawbones Europe AB, Malmo, Sweden) with implanted measuring stem embedded in gelatine was developed. The gelatine mixture was chosen in such a way that assembly forces applied to the model corresponded to those *in situ*. A study involving 31 surgeons was carried out on the aforementioned *in vitro* model, in which the assembly forces were determined.

**Results:**

A model was developed, with the influence of human soft tissues being taken into account. The assembly forces measured on the *in vitro* model were, on average, 2037.2 N ± 724.9 N, ranging from 822.5 N to 3835.2 N. The comparison among the surgeons showed no significant differences in sex (*P* = 0.09), work experience (*P* = 0.71) and number of THAs performed per year (*P* = 0.69).

**Conclusions:**

All measured assembly forces were below 4 kN, which is recommended in the literature. This could lead to increased corrosion following fretting in the head-neck interface. In addition, there was a very wide range of assembly forces among the surgeons, although other influencing factors such as different implant sizes or materials were not taken into account. To ensure optimal assembly force, the impaction should be standardized, *e.g*., by using an appropriate surgical instrument.

## Background

The replacement of a degenerated joint with an artificial joint is an extremely successful medical procedure, during which most patients regain their mobility within a short time and experience significant pain relief. The use of an artificial joint is therefore one of the standard surgeries in modern medicine today [1, 2]. Several hundred thousand hip endoprostheses are implanted worldwide each year [3–6], with sharp increase in demand [2, 6, 7].

Current THA systems are of modular design to provide the patient with the ideal clinical treatment. For example, the material of the bearing partners can vary or the anatomy of each individual patient can be taken into account. In hip endoprosthetics, virtually all implant manufacturers use the principle of a Morse taper junction.

However, in the recent past, there have been increasing reports concerning problems with taper junctions, which are usually accompanied by corrosion and abrasion. Though metal-on-metal bearings are especially affected [8–11], ceramic bearings are also affected [11, 12]. Such problems occur not only in primary [13] but also in revision THA [14].

Some studies examined the causes of failure. Explant studies have shown that impurities in the head-neck interface reduce its strength and increase the risk of corrosion [15]. Furthermore, the material [16, 17] and the assembly forces [17–19] seem to play an important role.

During implantation, the head-neck taper junction is joined by one or more hammer blows. The hammer has an impulse, which is partially or completely transmitted to the impactor tool and the femoral head upon impaction. The change in momentum of the hammer corresponds to the integral of the measured force over time. At the moment of impact, the hammer is abruptly decelerated (negatively accelerated). According to Newton’s Laws, the force is the product of mass and acceleration. Despite the relatively low mass of the hammer head, the high negative acceleration results in great forces of several kilonewtons for a short time. Decisive for the junction strength is the maximum force acting once in the direction of the neck axis [17].

However, it must be assumed that such a hammer blow is delivered very individually by the surgeon and is therefore not reproducible. This is illustrated by Nassutt *et al*. [20] who compared the assembly forces resulting from hammer blows of 39 surgeons. They showed that the force varied, ranging between 0.27 kN and 7.85 kN [20]. However, these trials were conducted on a test rig, so it must be assumed that the absolute values in surgery are different due to influence of soft tissues. Nevertheless, the study shows that the assembly forces could vary greatly among surgeons and that this influenced the strength of a Morse taper junction [17, 19]. The aim of this study was to investigate the assembly forces of different surgeons under *in situ* conditions.

## Methods

### Development of a suitable measurement system

**Fig. 1:**
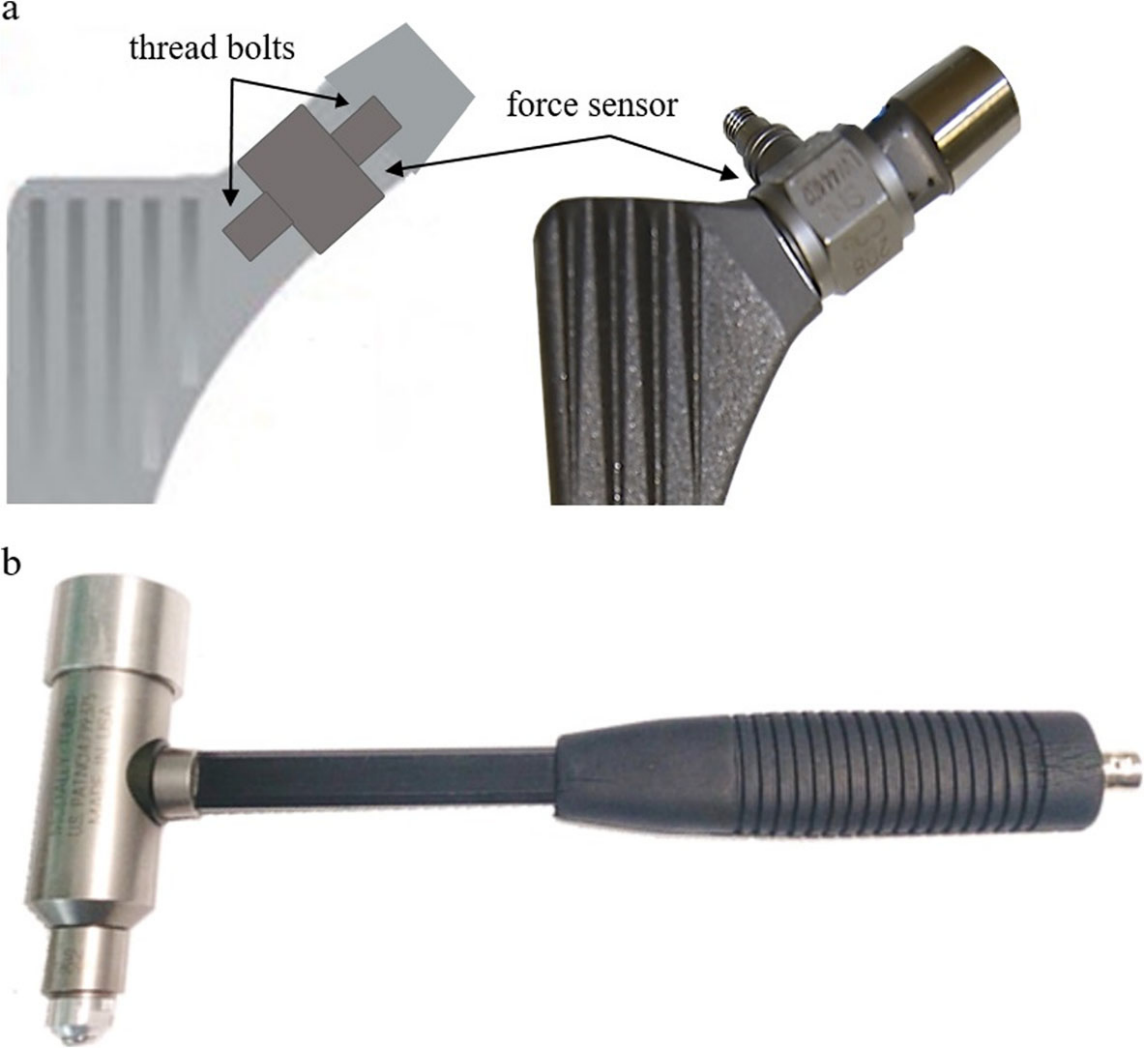
**(a)** Stem with integrated piezoelectric force sensor; (**b)** Impulse hammer

First, it was necessary to develop a measuring system capable of precisely measuring the highly dynamic forces occurring when head and the neck of the stem are being joined. For this purpose, piezoelectric sensors from PCB Piezotronics (Depew, NY, USA) were used. Piezoelectric sensors can measure high-frequency force signals and are available in small sizes. The housings of the sensors used are still hermetically sealed so they can be used *in situ*. A sensor (PCB 208C05) was integrated into the neck of a CBC Evolution stem from Mathys (Bettlach, Switzerland), as shown in Fig. 1(a). The sensor was mounted distally and proximally force-locked via thread bolts. Thus, it directly measured the force acting on the taper. Geometric parameters of the stem, like centrum-collum-diaphyseal angle or offset, were not changed by inserting the sensor. The used CBC Evolution is a standard non-cemented Spotorno stem and is laterally symmetrical, *i.e*., it can be implanted on both left and right sides. This allowed one stem to be used for both sides, which significantly reduced material costs.

Another component of the measuring system was an impulse hammer (PCB TLD086D05), which measured the forces on the impactor during the impaction. The hammer used is shown in Fig. 1(b). It was equipped with an additional weight in order to obtain the same weight (416 g) as a Bergmann mallet.

Both sensors were connected to a signal conditioner (PCB 482C15), which supplied the sensors and amplified the measuring signals. A Data Acquisition (DAQ) module with 16 bit A/D converters digitized each analogue signal at an 80 kHz sampling frequency. Afterwards, the digitized data were transmitted to a personal computer (PC), where the data were recorded by software specially developed in LabView (NI, Austin, USA).

### Experimental impaction on human cadavers

**Fig. 2:**
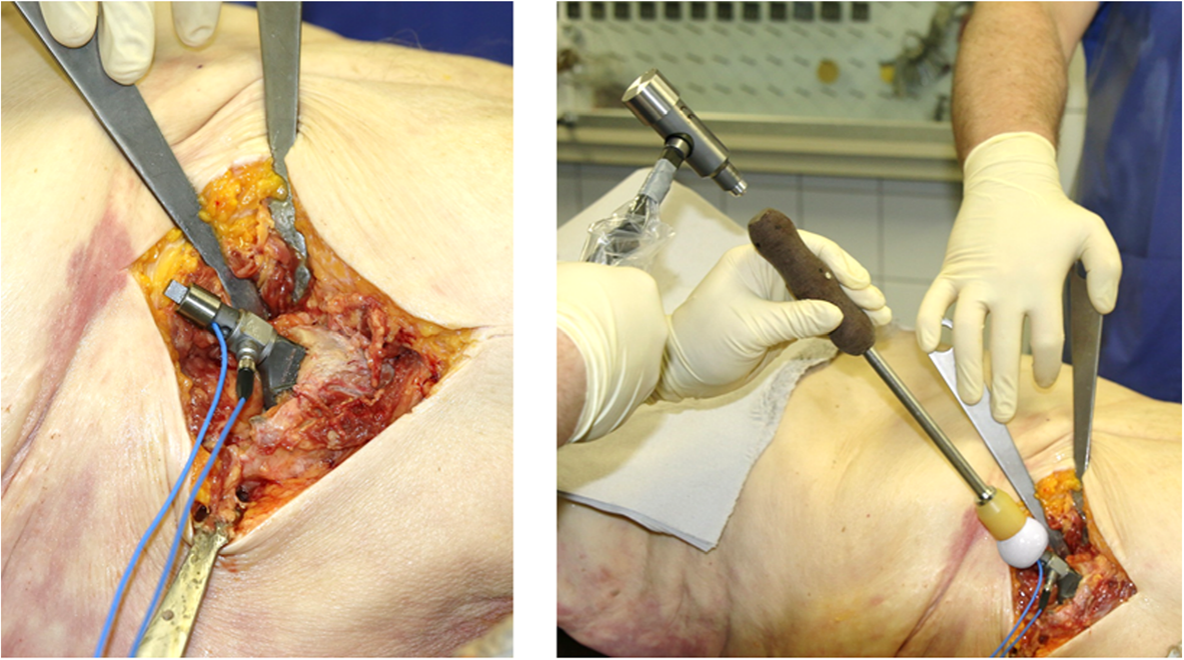
Left: Implanted measuring stem; right: Setup to measure *in situ* assembly forces

First, the developed measuring system was used to carry out a series of experiments on cadavers. For this purpose, the measuring prosthesis was implanted in chemically-untreated fresh, unfrozen human cadavers (Fig. 2, left). The use of fresh, unfrozen cadavers was important, because their soft tissues were very close to living humans in terms of mechanical properties. All implantations were performed by an experienced senior physician. The classic anterolateral approach according to Watson-Jones [21] was used to access the surgical site. The preparation was done in the muscle gap between tensor fasciae latae muscle and the gluteus group. An L-shaped opening of the ventral hip joint capsule was made. The dorsal parts of the capsule remained intact. The attachment of the gluteal muscle group at the greater trochanter was slightly loosened or notched in the distal portion. Other muscles or attachments, in particular, the iliopsoas muscle, were not injured or detached.

Due to the study structure and the short-term availability of the body donors at the Institute of Anatomy, no radiological data could be collected in advance, so planning based on X-ray images was not possible. The body donors always had to be cremated within 24 hours. Also, due to this procedure, the bone density could not be determined.

The ceramic head (ceramys, Ø 36 mm, size L, Mathys AG, Bettlach, Switzerland) was placed on the neck of the implanted stem and was then impacted by a hammer blow via the attached impactor tool (CBC head impactor, Mathys AG, Bettlach, Switzerland). The entire setup is shown in Fig. 2 (right). The resulting forces were recorded as described previously. Furthermore, the proximal femur was examined for possible fractures following impaction.

The procedure was performed on five hips from three different cadavers. One cadaver had an implanted femoral nail at the left side. In the case whose hips had not been subjected to previous surgical treatment, the measuring stem was implanted on both sides. The impaction was performed by three different surgeons. Surgeon I was an experienced senior physician, surgeon II a specialist physician and surgeon III an assistant physician. Surgeon I impacted three times and surgeon II and III impacted once each. The body donors were 71, 83 and 84 years old and weighed 54 kg, 65 kg and 92 kg, respectively. Since untreated fresh human cadavers were to be examined, the number of experiments was limited due to the small time window in which recruiting surgeons and carrying out the experiments were possible. In order to be independent of the restrictions associated with the use of cadavers and yet increase the number of experiments, the obtained data were used to develop an analogous *in vitro* model.

### Development of an *in vitro* model

**Fig. 3:**
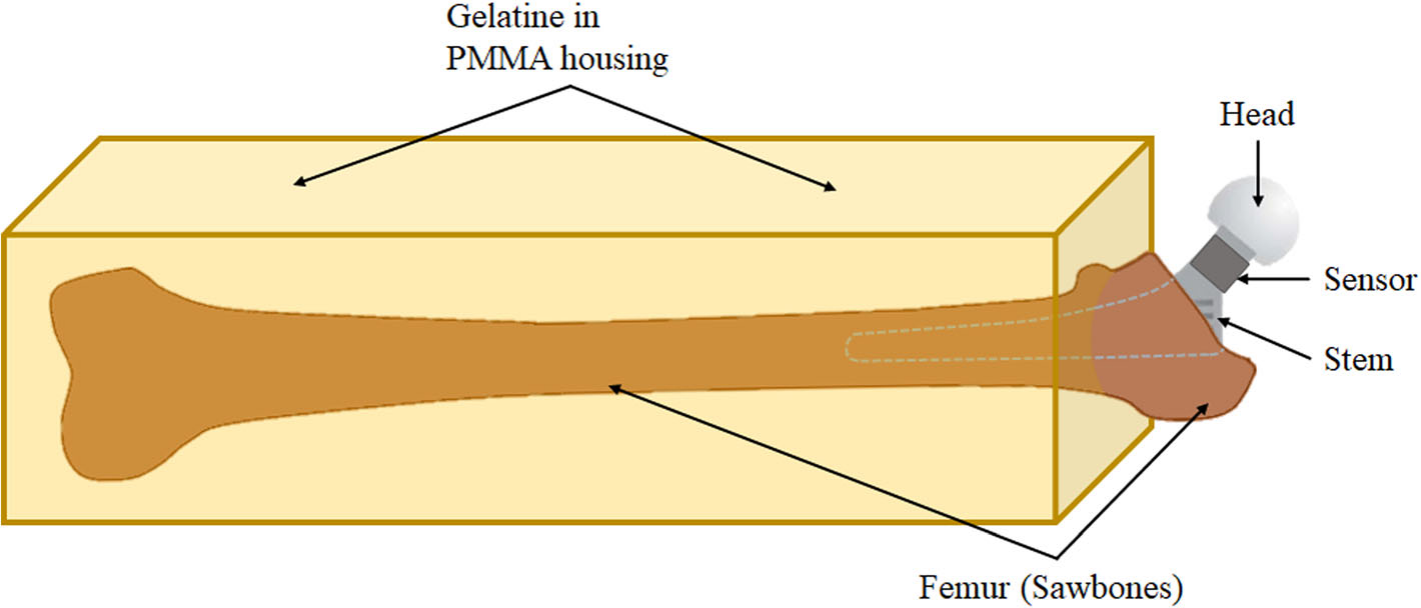
Schematic illustration of embedded femur with implanted THA stem

In order to obtain valid data using the analogous *in vitro* model, the main focus has been on the reproduction of the mechanical behaviours measured on the cadavers. Furthermore, the orientation and alignment of the setup played an important role. In particular, impact height or angles deviating from normal surgical procedures on the patient might falsify the measurement results. To meet these special requirements, the sensor-armed stem was first implanted into a 4th-generation artificial femur (Sawbones, Vashon Island, Washington; USA) by the same experienced surgeon as in the cadaver experiments. The femur was then embedded in a block of gelatine in such a way that the alignment and orientation of bone and stem corresponded to a THA with lateral transgluteal approach (Fig. 3).

**Fig. 4:**
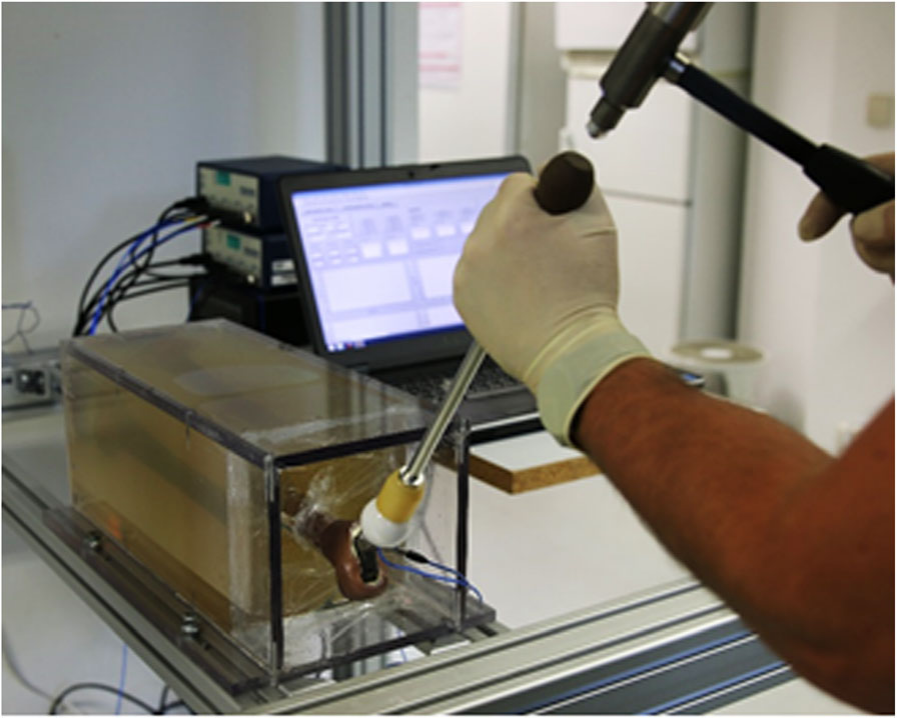
Final *in vitro* model

The gelatine block was designed to reproduce the mechanical behaviour of human soft tissues. It was cast in a housing made of polymethylmethacrylate (PMMA), which was rigidly screwed to a profile frame. As in the experiments on the cadavers, the sensors of the measuring system were connected to a signal conditioner and PC. The final *in vitro* model is shown in Fig. 4.

The mixing ratio of gelatine granulate and water determines the resulting stiffness of the gelatine. The aim was to produce the same properties as those present *in situ*. To find the optimal mixing ratio of the gelatine, the *in vitro* model was built with mixing ratios of 1:4 and 1:9 (gelatine granulate:water). As in the experiments on human cadavers, a ceramic head was placed on the neck of the stem and then impacted by a hammer blow. For each mixing ratio, 10 impactions were performed. The measured data were compared with those of the experiments on cadavers.

In order to quantify the mechanical behaviour of the model and cadaver and thus be able to compare them, a suitable parameter was needed. The ratio of applied impulse to peak force was considered as a possible parameter. The peak force is the maximal force that occurs during impaction and is also referred to as assembly force. The relationship between the peak force and the applied impulse is: the softer the system, the lower the peak force that occurs when the same impulse is applied. Consequently, systems have the same stiffness when the same peak force arises from the same impulse. Based on these considerations, the normalized peak force (NPF), which is the peak force divided by the applied impulse, was introduced as a suitable parameter for the comparison of the mechanical behaviours. Since only the maximum force during impaction is decisive for the taper junction, it was important for the model to reproduce the same peak force at the same impulse as in the experiments on the cadavers. The aim of the development of the *in vitro* model was not to reproduce the mechanical properties of all components quantitatively.

**Fig. 5:**
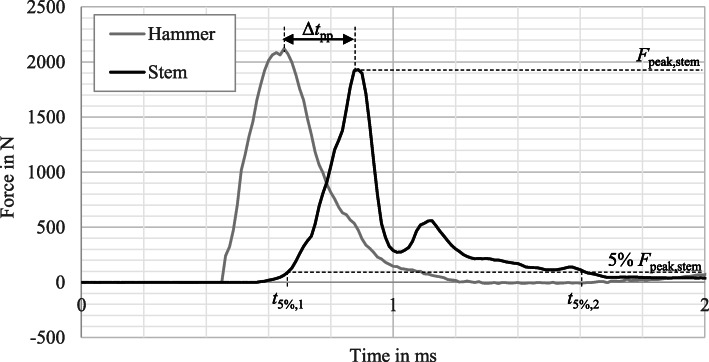
Force curves measured by sensors in hammer and neck of the stem while impaction

The impulse was calculated by integrating the force function between the points in time when the force exceeded or fell below 5 % of the peak force (*t*_5 %,1_ or *t*_5 %,2_). For further explanation, the stated parameters are shown in Fig. 5.

### Experimental impactions on *in vitro *model

A series of trials involving 31 surgeons has been carried out with the aforementioned *in vitro* model. First, each surgeon filled out an anonymous questionnaire containing the following information: sex, handiness, employment in the clinic, number of THA performed per year and the professional experience in years.

Each surgeon was instructed to assemble the head in the same way as they would do under normal intraoperative conditions. The only exception was the cleaning of the taper, because there was no contamination of the taper under laboratory conditions. In each case, the head was placed on the stem, the impactor tool was held against the head and then joined with a single hammer blow. Since these trials were carried out on the *in vitro* model, the damping behaviour was always the same. Thus, only the peak forces occurring in each case were evaluated as the decisive parameter for the strength of the connection.

Based on the information provided in the questionnaire (gender, professional experience and number of THAs per year), the surgeons were divided into different groups. Results of the groups were checked for normal distribution with the Shapiro-Wilk test. The normally-distributed mean peak forces achieved in the individual groups were then examined for statistically significant differences using the student *t*-test. A value of *P* < 0.05 was considered to be statistically significant. The calculations were performed using SPSS 24.0 (IBM, Armonk, New York, USA).

## Results

### Applied forces during experimental impactions on human cadavers

The force curves recorded by the two force sensors in hammer and the neck of the stem presented a similar course across all the trials as shown in Fig. 5 as an example. Both courses showed a rapid rise and fall in force. The pulse duration was always less than 1 ms. The force measured on the hammer was temporally slightly ahead of the force measured on the neck of the stem. This behaviour can be explained by the inertia of impactor tool and head, on the one hand, and by the distance which the head slides onto the taper, on the other. When the hammer hits the impactor tool, its inertia and that of the head counteract the inertia of the hammer. The force of the hammer accelerates the impactor tool and the head. As a result, the head slides on the taper and a force is built up at the sensor in the neck of the stem. The time offset between the two peaks Δ*t*_pp_ was 0.29 ms ± 0.14 ms.

Three different surgeons carried out impactions on three different human cadavers. The applied peak forces and impulses are listed in Table 1. None of the experiments resulted in fractures of the femur.

**Table 1:** Results of impactions on human cadavers

Body Donor			Side	Stem size	Surgeon	*F*_peak, hammer_ (N)	*I*_hammer_ (Ns)	*F*_peak, stem_ (N)	*I*_stem_ (Ns)
Sex	Age	Weight							
female	71	54	left	7	I	1733.0	0.448	1063.6	0.367
			right	7	II	2881.6	0.829	2453.4	0.679
female	83	65	left	8	III	1301.0	0.312	1127.4	0.291
			right	9	I	1449.2	0.510	1402.9	0.393
female	84	92	right	10	I	2122.1	0.592	1930.0	0.495

### Normalized peak forces at different mixing ratios

**Fig. 6:**
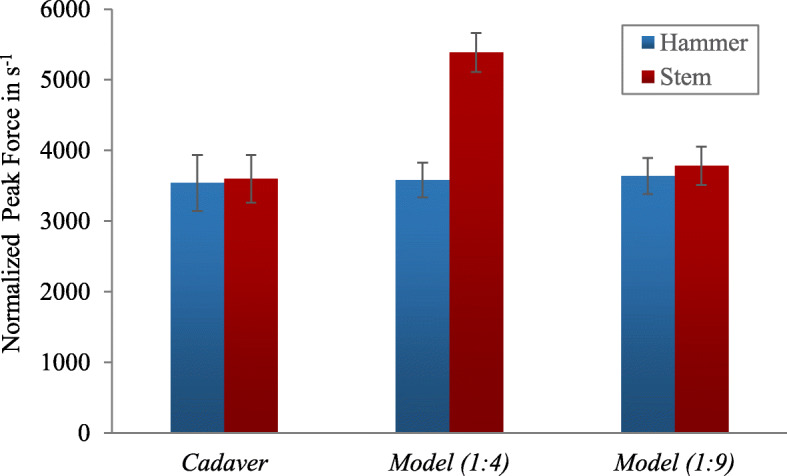
Box plot with determined normalized peak forces

The *in vitro* model was built at two different mixing ratios of the gelatine of 1:4 and 1:9. Experiments with 10 impactions each were carried out on both models. The NPF of the test series are shown in Fig. 6.

The NPF measured by the hammer showed no significant differences between cadavers and 1:4 model (*P* = 0.80) and between cadavers and 1:9 model (*P* = 0.56).

The NPF measured by the stem were significantly higher on 1:4 model than on the cadavers (*P* = 5.8*10^− 8^). In contrast, there were no statistically significant differences in the NPF between the cadavers and 1:9 model (*P* = 0.27).

**Fig. 7:**
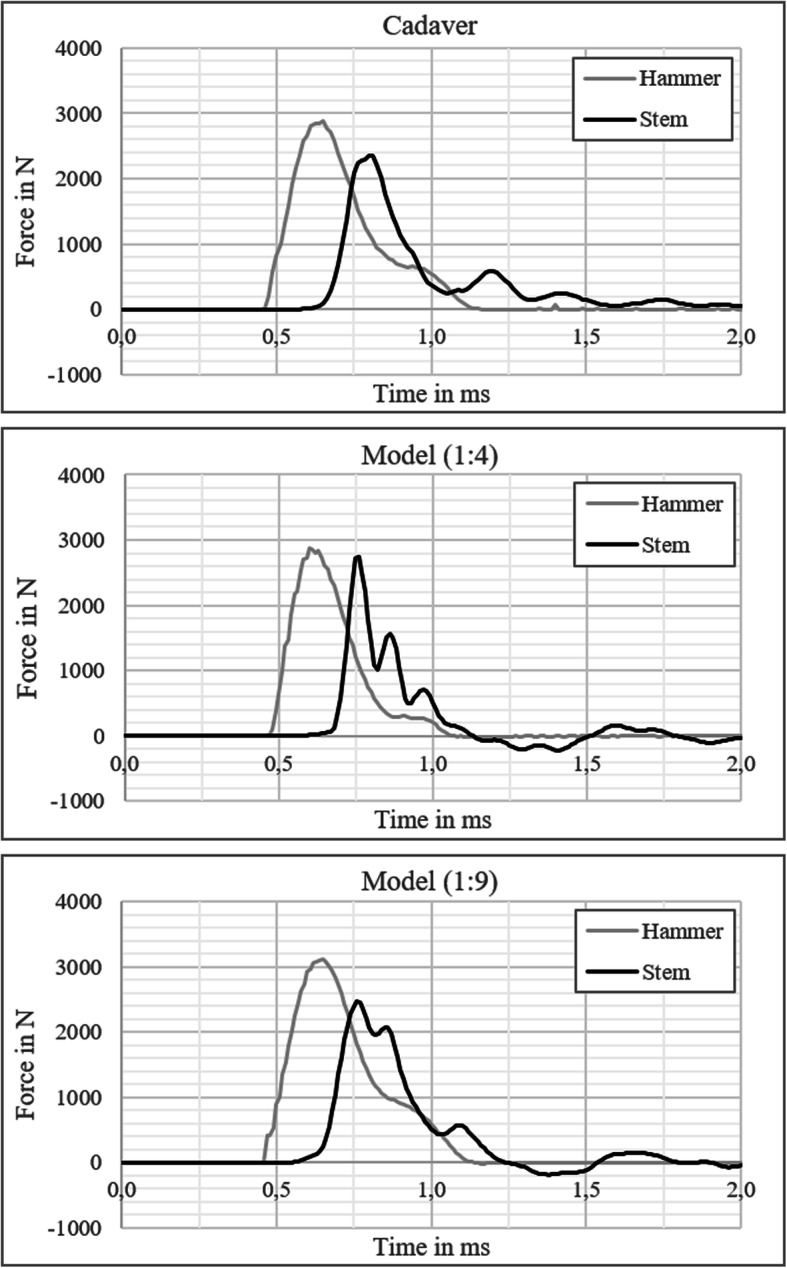
Exemplary force curves measured on (**a**) cadavers and on *in vitro* models with gelatine mixing ratios of (**b**) 1:4 and (**c**) 1:9

Furthermore, the exemplary force curves shown in Fig. 7 demonstrate the similarities and differences in the mechanical behaviour between the test setups. The force curve measured on stem on 1:4 model increased faster than on the other two curves and had almost the same peak force as on the hammer (*F*_peak, stem_ = 2747.6 N; *F*_peak, hammer_ = 2881.6 N). In contrast, the force curves measured on cadaver (*F*_peak, stem_ = 2453.4 N; *F*_peak, hammer_ = 2924.8 N) and 1:9 model (*F*_peak, stem_ = 3109.1 N; *F*_peak, hammer_ = 2463.9 N) show considerably lower peak forces on stem than on hammer.

Due to the similarity of the force curves and the NPF, the mixing ratio 1:9 was subsequently used for further experiments with the *in vitro* model.

### Applied forces during experimental impactions on *in vitro *model

**Fig. 8:**
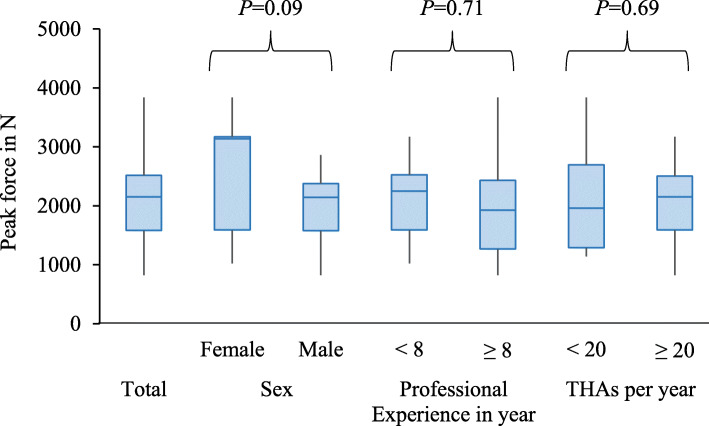
Box plot with determined peak forces in several groups of surgeons

Based on the evaluation of the questionnaires completed by the 31 surgeons, the groups shown in Fig. 8 were created. Five female and 26 male surgeons participated in the study. The average professional experience was 7.7 years. Therefore the participants were divided into surgeons with less than eight and equal to or more than eight years of professional experience. The group with less than eight years of professional experience had 17 and the group with 8 and more years had 14 surgeons. Another parameter collected was the number of THAs performed per year. 25 surgeons reported to perform less than 20 THAs per year and only six stated that they performed 20 or more. They were not classified in terms of the handedness, since only right-handed surgeons participated in the study.

The comparison of the groups using unpaired *t*-test showed no significant differences with regard to sex (*P* = 0.09), professional experience (*P* = 0.71) and number of THAs performed per year (*P* = 0.69). The assembly forces of all participants were on average 2037.2 N ± 724.9 N and ranged from a minimum of 822.5 N to a maximum of 3835.2 N.

## Discussion

The literature shows that the quality of the head-neck taper junction is influenced by many different factors. Firstly, contamination of the taper by blood or fat decreases the strength of the connection and should therefore be avoided [15]. Furthermore, damage to the taper, *e.g*. during revision surgery with head replacement, exerts a negative effect on the junction and can lead to head breakage in metal-ceramic bearings [22]. Therefore, an revision head with integrated metal sleeve should always be used in such cases [23]. Another decisive factor is the assembly force. If the assembly force is too low, there is an increased risk of corrosion at the interface due to fretting [24]. Too high assembly forces, on the other hand, can lead to proximal fractures of the femur, which then require extensive surgical treatment [25]. In contrast to the other factors described, there is no clinical standard for eliminating this potential risk. Therefore, the aim of this study was to determine the individual *in situ* assembly forces of different surgeons and to compare them with the forces recommended in the literature in order to derive a recommendation for action.

As the first step, experimental impactions were carried out on human cadavers. Since the sample size (*n* = 5) was very small due to the limited availability of body donors, an *in vitro* model was developed which reproduced the mechanical behaviour of the experiments on the cadavers. This *in vitro* model was intended to enable a larger number of trials. With the introduced parameter NPF, it could be shown that the ratio between the introduced impulse and peak force on the stem was almost identical in the trials on cadavers and on the *in vitro* model. Furthermore, the comparison of force curves measured on cadavers and on *in vitro* model showed high similarity. Therefore, it can be assumed that the mechanical behaviour was almost identical in both experiments. Reproducing physiological mechanical behaviours of human soft tissues *in vitro* not only allows the comparison of the assembly forces among different surgeons, but can also be used to determine the forces actually acting on the patient. For example, Scholl *et al*. [26] carried out a similar investigation on a very rigid structure and measured assembly forces up to approximately 25 kN. They explained the very high forces, as compared to other studies [17], resulted from the different point where the force was measured (striking pad of the hammer). In this context, it should be noted that the participating surgeons believed the striking pad of the hammer used to be too small, which can be seen as a limitation of the experiments. Nevertheless, this study showed that such high forces did not occur neither on the hammer nor on the stem under physiological damping behaviours on the patient. The maximum forces measured on the hammer were 2881.6 N on cadavers and 4058.3 N on the model. The measured forces on the hammer were slightly greater than those measured on the stem. This is ascribed to different bearing situations of impactor tool and stem, friction and also differences between impact direction of the hammer and the neck axis.

However, the difference accounted for only a few percent. Krull *et al*. [27] also carried out experiments on an *in vitro* model in the laboratory. They showed that both the stiffness of the tip of impactor tool and the bearing of the taper have major influence on the resulting forces. Thus, the bearing is a much more significant influencing factor than the point of measurement. In this study, the focus was on the forces measured on the neck of the stem. The force acting on the neck of the stem is the force that counteracts the impact of the hammer and thus the force that is decisive for the Morse taper junction.

As already shown in similar studies, there are considerable differences in the assembly forces among individual surgeons [20, 26]. The tests on cadavers yielded forces between 1063.9 N and 2453.4 N. Even larger differences occurred when testing on the *in vitro* model due to the greater number of cases. The forces ranged from a minimum of 822.5 N to a maximum of 3835.2 N. As the findings showed, the range did not depend on sex, age or experience of the surgeons.

Based on the results of other studies, which examined the effects of assembly forces on parameters as pull-out force, turn-off moments and fretting, it appears that most of the surgeons involved applied too little force. Based on the resulting pull-out forces from different assembly forces, Ramoutar *et al*. recommend a minimum assembly force of 2.5 kN [19]. Consequently, 71.0 % of the surgeons would not have applied a sufficiently high assembly force. On the basis of their findings regarding the relation between assembly force and resulting turn-off moments, Rehmer *et al*. recommended an assembly force of 4 kN [17]. From their investigations on fretting, Haschke et al. also recommended an assembly force of 4 kN [28]. According to this demand, 100 % of the surgeons showed insufficient assembly forces in this study. However, Rehmer *et al*. [17] and Haschke *et* *al*. [28] also recommend that assembly forces of more than 4 kN be avoided, or the risk of intraoperative proximal fractures of the femur increases.

In conclusion, the assembly forces determined showed a high variance among surgeons and they were low compared to the data given in the literature. The influences of different head sizes, material combinations or different soft tissue conditions were ignored in this study. This should be taken as a limitation of the study. It can be assumed that the actual variance of the assembly forces might be even higher. Heads of different sizes and especially different materials differ in weight and different material combinations of head and stem have different friction properties at the interface [29]. These influencing factors could be investigated in further studies using the *in vitro* model developed.

In our opinion, it is highly recommended to standardize the impaction of the femoral head by using a new type of surgical instrument.

## Conclusions

The assembly force generated by the surgeon depends on many different factors. One very decisive factor is the damping behaviour of the patient’s soft tissues. The tests performed on the *in vitro* model, which was built based on the actual soft tissue situation, showed that surgeons applied too little forces to achieve an optimal head-neck taper junction. Because of the inherent risk of intraoperative femur fractures when the assembly force applied is too high, surgeons should have an aid, such as a surgical instrument, to ensure that the correct force is constantly applied. When measuring the assembly forces, we believe it is important to measure below the taper, since this is where the force occurs and that is decisive for the strength of the Morse taper junction.

## Data Availability

The datasets used and analysed during the current study are available from the corresponding author on reasonable request.

## Abbreviations

THA: Total hip arthroplasty
OP: Operation
NPF: Normalized peak force
DAQ: Data acquisition
PC: Personal computer
PMMA: Polymethylmethacrylate
